# Analysis of electroencephalography brain rhythms in the reading process

**DOI:** 10.31744/einstein_journal/2020AO5442

**Published:** 2020-10-29

**Authors:** Camila Davi Ramos, Izabella Nonato Oliveira Lima, Amanda Luiza Rodrigues, Kaliny Alice Carvalho de Oliveira Magalhães, Aurélia Aparecida de Araújo Rodrigues, João-Batista Destro-Filho

**Affiliations:** 1 Universidade Federal de Uberlândia UberlândiaMG Brazil Universidade Federal de Uberlândia, Uberlândia, MG, Brazil.

**Keywords:** Electroencephalography, Reading, Gamma rhythm, Delta rhythm, Beta rhythm

## Abstract

**Objective:**

To verify if, by three distinct quantifiers, the measured electroencephalographic signal at rest is different from the signal measured during a word reading situation, especially considering the faster rhythms, gamma and high-gamma, as it occurs in clinical rhythms (delta to beta).

**Methods:**

A total of 96 electroencephalographic signals measured from neurologically healthy volunteers were evaluated at two moments: resting and word reading. Each signal segment was measured by three quantifiers that separately assess normalized power, percent power, and right and left hemisphere coherence. The Mann-Whitney test was used to compare the results of the quantifiers in each brain range.

**Results:**

The gamma and high-gamma rhythms presented a more distinct behavior when comparing the analyzed moments (resting and reading) than the clinical rhythms.

**Conclusion:**

This finding contributes to the scarce literature on faster rhythms, which can contain information that is normally disregarded in neurological clinical practice.

## INTRODUCTION

Reading improves the storage of information in the human brain, and requires a combination of skills and continuous study to enhance knowledge by learning of new words.^(^^1^^)^ Multiple brain systems must interact cooperatively for a word to be recognized and a homogeneous interpretation of the visual input to occur.^(^^2^^)^ Research indicates that several brain regions are triggered during reading.^(^^3^^)^ However, using neuroimaging, it has been shown that an area in the occipitotemporal cortex is activated in fluent readers.^(^^4^^)^ This region, located in the fusiform gyrus, has been termed the visual word form area.^(^^5^^)^ Studies showed the activation of this region is evoked by written words, but not by spoken words.^(^^5^^)^ Besides improving previously acquired knowledge, reading also improves auditory and visual perception,^(^^6^^)^ and is a particular form of human evolution.

During a word reading situation, the electrical activity of the brain changes, and these changes can be captured in electroencephalogram (EEG) recordings. The transmission of brain information occurs via electrical activity, and the EEG records this activity, which is detected by electrodes placed on the scalp,^(^^7^^,^^8^^)^ according to the following neuroanatomical locations: frontal (F), central (C), parietal (P), temporal (T), occipital (O). Odd numbered electrodes represent the left cerebral hemisphere, and even numbered electrodes represent the right hemisphere.^(^^9^^)^

The EEG spectral analysis is performed in the EEG frequency bands, which have well-defined limits,^(^^9^^,^^10^^)^ namely: delta (ranging from 0.5 to 3.5Hz), theta (from 3.5 to 7.5Hz), alpha (ranging from 7.5 to 12.5Hz), beta (from 12.5 to 30Hz), gamma (from 30 to 80Hz), and high-gamma (above 80Hz).^(^^10^^,^^11^^)^ The literature reports that the gamma and high-gamma rhythms are related to cognitive tasks involving perception, attention and memory.^(^^12^^,^^13^^)^ However, as far as the authors are aware, no study analyzed the reading process per se. All previous studies analyzed reading associated with word comprehension, text interpretation, and visual stimulation. In addition, few articles studied the reading process in frequency bands above 40 Hz,^(^^1^^,^^4^^,^^9^^,^^14^^)^ particularly in the context of Brazilian or Latin American individuals, assessing the degree of quantitative EEG changes that occur between resting and reading states, which is extremely important for a correct calibration of a brain-machine interface.

## OBJECTIVE

To measure, by quantitative processing, the electroencephalographic signals of neurologically healthy volunteers during a word reading situation and in a resting situation, and to verify if the gamma and high-gamma rhythms contribute significantly to the differentiation between the reading and the resting states, demonstrating possible changes in brain activity caused by these rhythms in these two situations.

## METHODS

This study was carried out in the first semester of 2019 and addressed the following topics in its methodological process: data collection; pre-processing; quantification of the brain signal; and statistical analysis.

### Data collection

We analyzed EEG signals considered neurologically normal, collected from an EEG database and recorded from healthy volunteers, between the years 2016 and 2018.^(^^15^^)^ Authorization by the Research Ethics Committee of the *Universidade Federal de Uberlândia* (UFU) was released under CAAE: 54781615.6.0000.5152, with approval number 1.715.960, allowing the collection and use/processing of these data. From this database, 96 EEG records were used (49 of the records were from male volunteers). The mean age of the volunteers was 24±7.5 years.

We used a 20-channel EEG equipment, with a high-pass filter with a cut-off frequency of 1Hz, a 100Hz low-pass filter, and a filter that rejected the range between 58 and 62Hz. The sampling frequency and impedance values set for the device were 1kHz and 1 KΩ, respectively. All records were collected following the international 10-20 collection system. The protocol recorded two events: event 1, termed no stimulus (NS) event, and event 2, in which the volunteer read ten words sequentially, termed word reading (WR) event.

**Figure 1:**
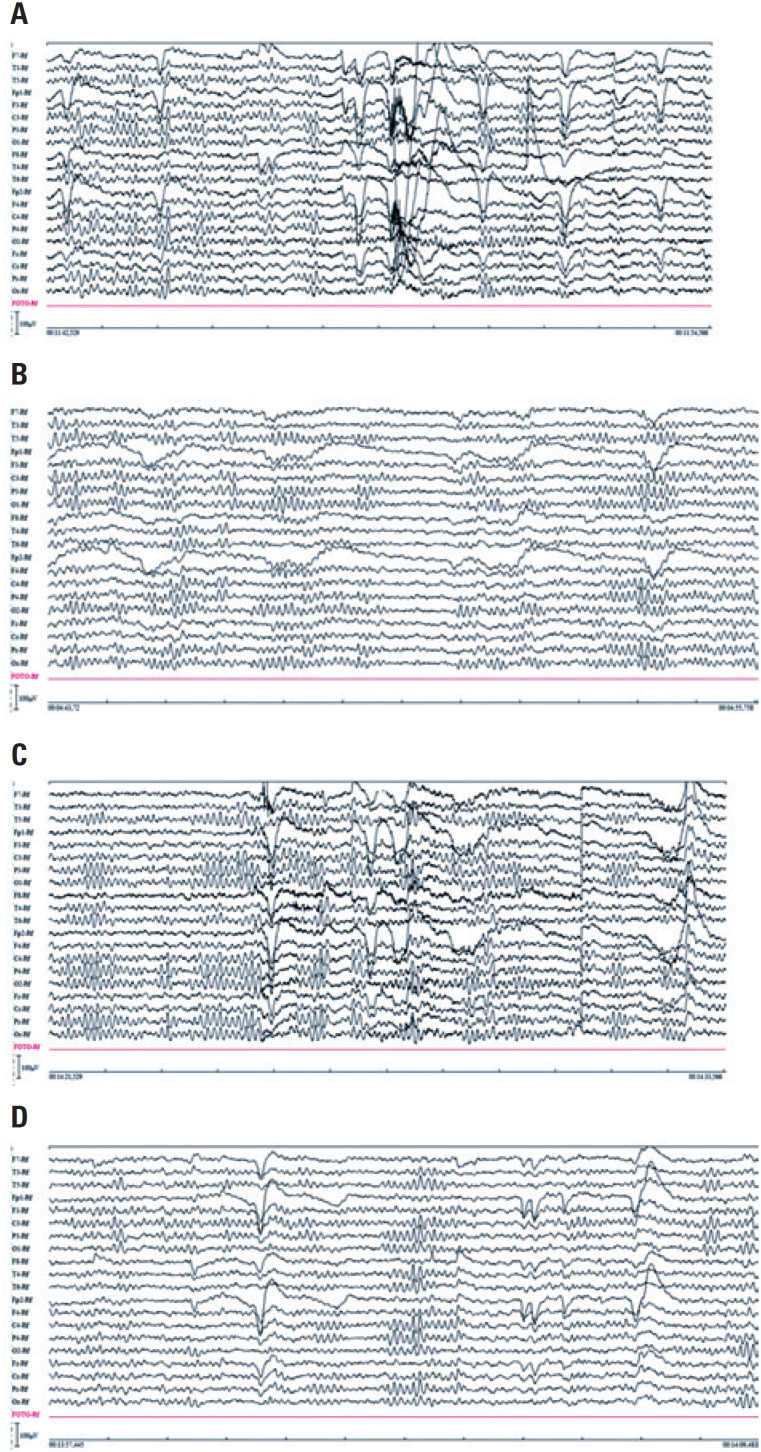
Examples of unfiltered and filtered electroencephalographic signals in the time domain collected from a volunteer. (A) unfiltered signal of the no stimuli situation; (B) filtered signal of the no stimuli situation; (C) unfiltered signal of the word reading situation; (D) filtered signal of the word reading situation

For NS recording, the volunteer laid down, with eyes open and at rest, for 3 minutes, without any stimulation. In WR, the volunteer laid down, and one word at a time was presented sequentially. Each word was shown for a period of 5 seconds, at the height of the volunteer's eyes, written in large fonts on a piece of cardboard, for a silent reading. After the reading, another 5 seconds of recording took place, in silence, before moving on to the next word. In total, the volunteer was asked to read ten words, namely: dog, football, shirt, soap opera, banana, tomato, music, cell phone, fabric and work. This choice of words, although random, sought to use words that evoked memories and emotions associated with the daily life of a common Brazilian individual. Examples of filtered and unfiltered EEG signals in the time domain, collected from a volunteer for both situations studied here, are shown in figure 1.

### Pre-processing

To ensure that the study considered a time interval during which the volunteer was actually performing the word reading act, the first and the last seconds recorded for each word read were excluded. Thus, for the WR event, ten 3-second epochs (stretches and segments) were analyzed, each epoch associated with the reading of a specific word. As for the NS event, each EEG recording was validated for separation into epochs of interest. The epochs, also lasting 3 seconds, were selected by a neurologist, who was a specialist in EEG analysis, through visualization. The morphology of a neurologically normal recording, devoid of visual artifacts, as expected for a healthy volunteer, was adopted as pattern.

We relied on filtering methods and visual analysis to avoid artifacts, such as 60Hz noise, muscle artifacts, blinks, or others, to assure that the analyzed signal was related to the EEG tracing.

### Quantification of the brain signal

The first tool used to quantify the signal was the discrete time Fourier transform (DTFT), whose calculation results in the spectral density (power) of the signal distributed at each frequency analyzed. The DTFT calculation is defined as shown in equation 1.

(Equation 1) $$F(\omega )=\int_{-\infty }^{\infty }f(t)e^{-i\omega t}dt$$

where F(ω) is the DTFT of f(t).

The normalized power percentage (NPP) quantifier measures the amount of signal power, covering all existing rhythms between 1 and 100Hz (delta, theta, alpha, beta, gamma and high-gamma). The power contribution percentage (PCP) quantifier measures the power of each brain rhythm relatively to the total power of the spectrum, informing the contribution of each rhythm in the evaluated epoch. The input data for both quantifiers were ten epochs of the NS event, and ten epochs of the WR event.

Initially, for both NPP and PCP, the power spectral density *S_x_*, was calculated using the DTFT.^(^^15^^–^^17^^)^ With the result of the calculation *S_x_*, for all epochs of the NS and WR events, the values of NPP (equation 2), and the values of PCP were calculated (equation 3).^(^^18^^)^ The NPP quantifier is defined as in equation 2.

(Equation 2) $$PPN_{x_{a}}(f,b)=\frac{S_{x}a(f,b)}{max\left(S_{x}a\right)}$$

Where S_x_a(f,b) is the power spectral density of the EEG signal at the X electrode, for event *a* (NS or WR); where *b* and *f* correspond, respectively, to the corresponding epoch and the frequency value. S_x_a is the power spectral density matrix, in which the lines represent the frequency values (f), and the columns refer to the epochs (b) [W/Hz]. Max(S_x_a) is the maximum amplitude, calculated after analyzing all elements of the S_x_a matrix [W/Hz].

In this way, the calculation of the PCP quantifier is made according to equation 3:

(Equation 3) $$PCP_{rhythm}i=\frac{\int_{f=f_{in}rhythm}^{f_{out}rhythm}{\left|S_{x}i_{a}(f)\right|}^{2}df}{P_{i_{a}}}$$

Where the index *i* is associated with the epochs; index *a*, with the event (NS or WR); *f*_in_*rhythm* is the first frequency value of the analyzed rhythm [Hz]; *f*_out_*rhythm* is the last frequency value of the analyzed rhythm [Hz]; *Pi*_a_ is the power calculated across the spectrum [W], whereas *a* is the event.

The input data for the coherence quantifier were the same as for NPP and PCP (selected epochs of each event). Coherence is a statistical measure related to the degree of coupling of the frequency spectra, and its value depends on the correlations between the events in the frequency domain. Additional diagnostic information can also be provided by measuring the pairs of neocortical regions.^(^^18^^–^^20^^)^ This refers to the degree of phase similarity between two signals.^(^^21^^)^ The coherence calculation is done by the square magnitude of the cross spectral density of the two signals, as seen in equation 4.

(Equation 4) $${\left|{\text{Γ}}_{xy}\left(e^{jw}\right)\right|}_{\;\;b}^{2}=\frac{{\left|s_{xy}\left(e^{jw}\right)\right|}^{2}}{S_{x}\left(e^{jw}\right)S_{y}\left(e^{jw}\right)}$$

I *b* is the epoch considered; S_xy_, the cross power spectral density [W/Hz]; S_x_, the spectral density of the X electrode; and S_y_, the spectral density of the U electrode.

Results of equation 3 vary from zero to one, and values close to zero indicate a low correlation, and values close to one indicate a high correlation. This property is expressed by equation 5.

(Equation 5) $$0\leq {\left|{\text{Γ}}_{xy}\left(e^{jw}\right)\right|}^{2}\leq 1$$

To perform the coherence calculations, the electrode pairs F3–F4, C3–C4, P3–P4 and O1–O2 were taken into consideration, in the spectral range from 1 to 100Hz, from the delta rhythm to the high-gamma rhythm.

### Statistical analysis

Since the NPP values varied over a wide range of frequency bands (1 to 100Hz), it was decided to divide these data into three bands: clinical rhythms (ranging between 1 and 30Hz); gamma rhythm (from 30 to 80Hz); and high-gamma rhythm (from 80 to 100Hz). From the NPP values per band, the NS and WR events were arranged in that order, so that time-frequency diagrams could be drawn up. These values were arranged in chronological order of events and by frequency, from the lowest to the highest values (considering each band separately). For each electrode analyzed, three time-frequency diagrams were generated: clinical rhythms, gamma rhythm, and high-gamma rhythm.

In order to verify the hypothesis of equality of NPP data in both events (NS and WR), the Mann-Whitney test was used, with 95% confidence interval. This is a non-parametric test for comparing independent samples to verify whether there is a difference between them.^(^^22^^)^ The result shows the level of significance between the data, represented by the p value. When the p value is less than 5%, the equality hypothesis is rejected, assuming that the compared data are different.

The PCP values obtained were assessed using descriptive calculation (median±median standard deviation) to summarize the information obtained from each brain rhythm. These values were also compared for the two events using the Mann-Whitney test with the same level of significance, and the results are shown in a table.

Finally, the coherence values obtained in both the NS and WR events were used to create time-frequency diagrams. The time axis was defined, as well as for NPP, by values measured in the chronological epochs, respecting the order of events, in seconds, and the frequency axis comprised values ranging from the lowest to the highest contribution within each rhythm, in Hz. The statistical comparison between the coherence values measured in the NS event and those obtained in the WR event was also implemented using the Mann-Whitney test, with a 5% significance level.

## RESULTS

**Figure 2:**
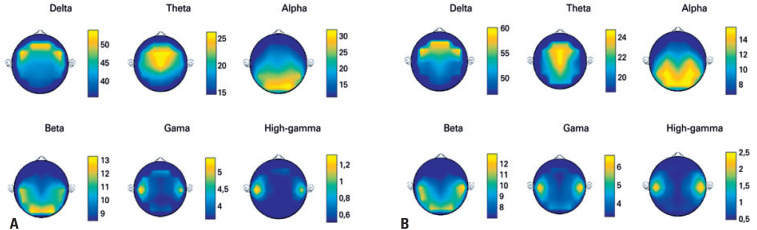
Topographies of the mean values of power contribution percentage of all volunteers. (A) topographies of clinical rhythms (delta to beta), gamma, and high-gamma for the no stimulus situation; (B) topographies of clinical rhythms (delta to beta), gamma, and high-gamma for the word reading situation

Figure 2 shows non-normalized topographies with the mean values of PCP of all volunteers for all rhythms: clinical (delta to beta), gamma, and high-gamma, demonstrating how the distribution and variation of rhythms occur, during the resting situation and the word reading situation, throughout all scalp leads. Using non-normalized topography, each rhythm has its own scale, which is constructed using the maximum and minimum average power of the specific rhythm.

**Figure 3:**
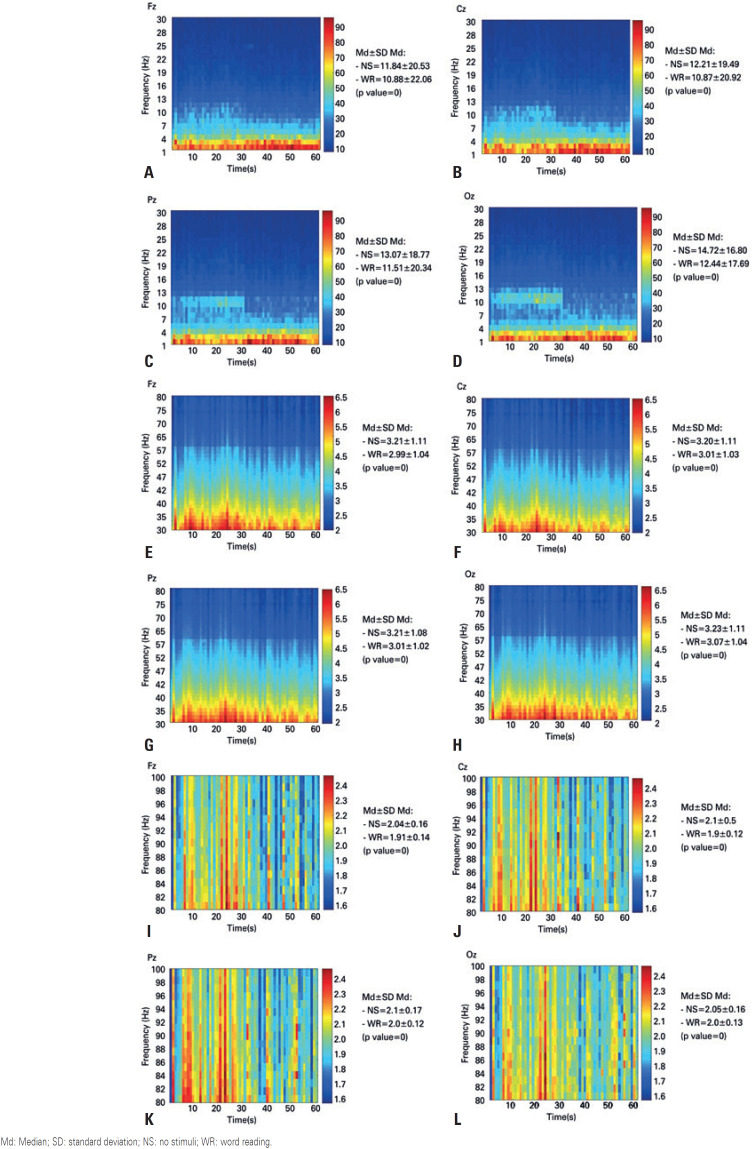
Time-frequency diagram of normalized power percentage for brain rhythms, with the first 30 seconds referring to the no stimulus event, and the last 30 seconds referring to the word reading event. (A) diagram of the Fz electrode, clinical rhythms; (B) diagram of the Cz electrode, clinical rhythms; (C) diagram of the Pz electrode, clinical rhythms; (D) diagram of the Oz electrode, clinical rhythms; (E) diagram of the Fz electrode, gamma rhythm; (F) diagram of the Cz electrode, gamma rhythm; (G) diagram of the Pz electrode, gamma rhythm; (H) diagram of the Oz electrode, gamma rhythm; (I) diagram of the Fz electrode, high-gamma rhythm; (J) diagram of the Cz electrode, high-gamma rhythm; (K) diagram of the Pz electrode, high-gamma rhythm; (L) diagram of the Oz electrode, high-gamma rhythm Md: Median; SD: standard deviation; NS: no stimuli; WR: word reading.

Figure 3 shows the time-frequency diagrams of the NPP values. In each figure, the Fz, Cz, Pz and Oz electrodes are shown. Figure 3, from A to D, shows the clinical rhythms; from E to H, shows the gamma rhythm; and from I to L, shows the high-gamma rhythm. In all comparisons between NS (1 to 30 seconds) and WR (30 to 60 seconds) using the Mann-Whitney statistical test, the p values were less than 0.05, indicating a statistical difference between the NPP measured at rest and the NPP measured during the word reading situation.

Figure 3, from A to D, shows that, in the frequency range between 10 and 13Hz, which the alpha band was more evident, the discrepancy between the NS and WR events was noticeable. In figure 3, from E to H, showing the gamma rhythm, the highest NPP values were concentrated in the 30 to 52Hz bands, not only visually, but quantitatively. These values decreased in the WR process (p value <0.05). In figure 3, from I to L, showing the high-gamma rhythm, there was a more evident separation between NS and WR in this frequency range, and also, as in the gamma rhythm, there was a decrease in the NPP values during the WR event.

The PCP values of each event, with their respective p values, for each brain rhythm, are shown in table 1 (delta to beta) and table 2 (gamma and high-gamma), for the C3, Cz, C4, P3, Pz, P4, T5, T6, O1, Oz, and O2 electrodes.

**Table 1:** Percentage values of power contribution – clinical rhythms (α=5%)

Electrodes and situation		Delta	Theta	Alpha	Beta
C3					
	NS	40.9	19.7	11.2	9.1
	WR	51.3	18.8	7.8	7.6
	p value	0	0.6	0	0
CZ					
	NS	40.8	23.3	11.4	8.2
	WR	53.3	22.4	7.5	6.6
	p value	0	0.3	0	0
C4					
	NS	41.1	18.3	12.9	9.2
	WR	52.9	18.2	7.6	7.4
	p value	0	0.8	0	0
P3					
	NS	40.7	18.3	13.1	9.3
	WR	49.9	18.3	8.7	8.2
	p value	0	0	0	0
PZ					
	NS	41.5	16.0	13.2	9.1
	WR	52.6	19.7	8.5	7.8
	p value	0	0	0	0
P4					
	NS	40.2	14.7	14.7	9.2
	WR	50.8	18.5	8.9	8.3
	p value	0	0	0	0
T5					
	NS	39.9	15.6	13.3	9.8
	WR	49.5	17.6	8.6	9.8
	p value	0	0	0	0.5
T6					
	NS	39.9	13.2	14.4	9.6
	WR	51.3	17.0	8.4	9.1
	p value	0	0	0	0
O1					
	NS	35.7	12.4	15.3	11.1
	WR	47.9	18.4	9.6	9.9
	p value	0	0	0	0
OZ					
	NS	37.1	13.1	15.2	10.5
	WR	48.8	17.9	9.9	9.6
	p value	0	0	0	0
O2					
	NS	35.3	10.9	18.2	11.0
	WR	47.8	18.1	10.4	10.1
	p value	0	0	0	0

p≥0.05 indicates statistical equality.

NS: no stimulus; WR: word reading.

**Table 2:** Percentage values of power contribution – faster rhythms (α=5%)

Electrodes and situation		Gamma	High-gamma
C3			
	NS	3.8	0.5
	WR	3.5	0.4
	p value	0	0
CZ			
	NS	3.5	0.4
	WR	3.1	0.4
	p value	0	0
C4			
	NS	3.7	0.5
	WR	3.0	0.4
	p value	0	0.1
P3			
	NS	3.6	0.5
	WR	3.6	0.5
	p value	0.7	0.7
PZ			
	NS	3.5	0.4
	WR	3.3	0.4
	p value	0.1	0.1
P4			
	NS	3.5	0.5
	WR	3.7	0.5
	p value	0.1	0.3
T5			
	NS	3.8	0.5
	WR	4.3	0.5
	p value	0	0
T6			
	NS	3.6	0.5
	WR	4.1	0.5
	p value	0	0
O1			
	NS	3.7	0.5
	WR	4.1	0.5
	p value	0	0
OZ			
	NS	3.6	0.5
	WR	3.9	0.5
	p value	0	0
O2			
	NS	3.7	0.5
	WR	4.1	0.5
	p value	0	0

p≥0.05: indicates statistical equality.

NS: no stimulus; WR: word reading.

In the PCP calculation, as to clinical rhythms, the delta rhythm showed an increase in power in all electrodes during WR, when compared to NS. On the other hand, both in alpha and beta rhythms, PCP values decreased in the word reading situation. The theta frequency band variation showed a different PCP behavior, according to the region measured. For example, in the P, O and T regions, the PCP increased during the word reading situation, whereas in the other regions this index decreased. In gamma and high-gamma rhythms, PCP values decreased in the word reading situation, except in the occipital and temporal region. The gamma and high-gamma rhythms, in terms of PCP, were sensitive to the brain region. In the parietal region, there was no differentiation between the PCP values measured at rest and during the word reading situation. This indicated that, for these rhythms, analyzing the electrodes in this region was not effective for EEG segmentation into resting and cognitive activity.

**Figure 4:**
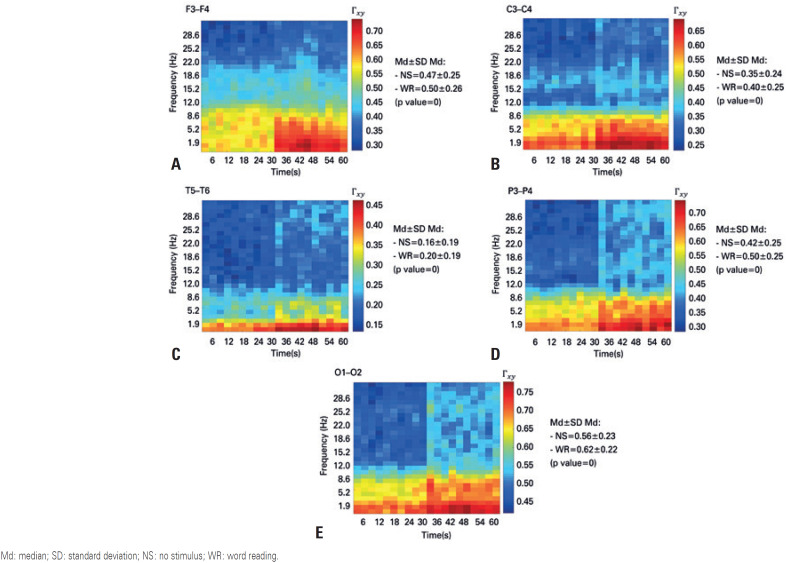
Time-frequency coherence diagram for clinical rhythms, with the first 30 seconds referring to the no stimulus event, and the last 30 seconds referring to the word reading event. (A) diagram of the Fz electrode; (B) diagram of the Cz electrode; (C) diagram of the Pz electrode; (D) diagram of the Oz electrode Md: median; SD: standard deviation; NS: no stimulus; WR: word reading.

**Figure 5:**
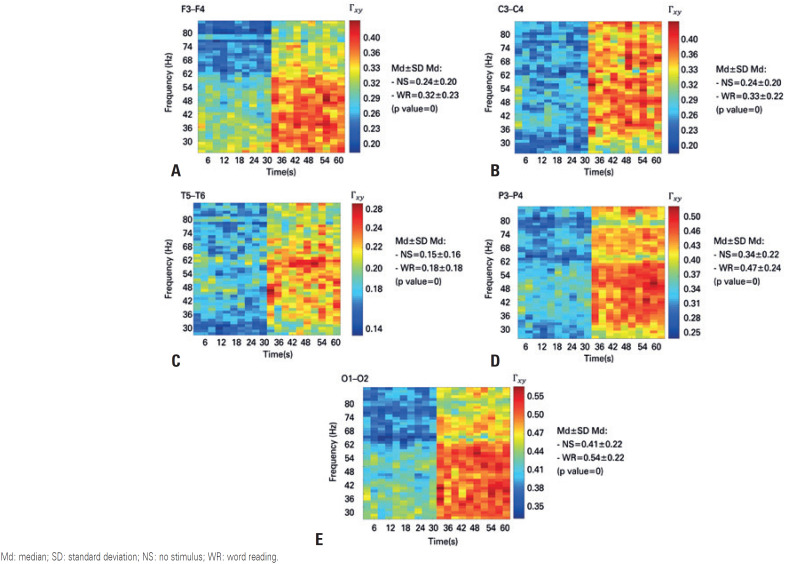
Time-frequency coherence diagram for gamma band, with the first 30 seconds referring to the no stimulus event, and the last 30 seconds referring to the word reading event. (A) diagram of the Fz electrode; (B) diagram of the Cz electrode; (C) diagram of the Pz electrode; (D) diagram of the Oz electrode Md: median; SD: standard deviation; NS: no stimulus; WR: word reading.

**Figure 6:**
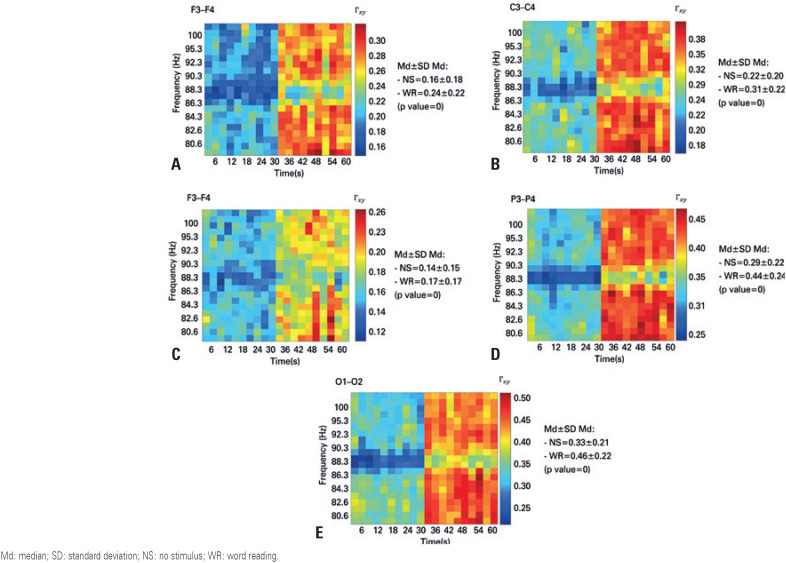
Time-frequency coherence diagram for high-gamma band, with the first 30 seconds referring to the no stimulus event, and the last 30 seconds referring to the word reading event. (A) diagram of the Fz electrode; (B) diagram of the Cz electrode; (C) diagram of the Pz electrode; (D) diagram of the Oz electrode Md: median; SD: standard deviation; NS: no stimulus; WR: word reading.

The analysis of the third EEG signal quantifier, *i.e*. coherence, indicated that both for the clinical rhythms (Figure 4) and the gamma and high-gamma rhythms (Figures 5 and 6, respectively), the coherence between the symmetrical pairs of the electrodes was higher during the word reading situation. The discrepancy between these values measured at rest and during word reading situation was markedly greater for faster rhythms, rendering these rhythms clinically important in the evaluation conducted by neurologists.

## DISCUSSION

The review by Antonenko et al.,^(^^23^^)^ describes two case studies: one on the cognitive load in hypertext reading, with 20 students, and the other on the cognitive load in multimedia reading, with 38 students. The results of the first case showed a significant decrease in alpha power during the 20 seconds of reading and, in the second case, this reduction was also noticed in the frontal region of the brain.

In the study by von Stein et al.,^(^^20^^)^ with 19 volunteers, coherence was analyzed in three different modalities: written word, auditory presentation and visual presentation of the object. Each stimulus lasted 2 seconds, with an interval of 2 to 3 seconds between all conditions. For comparison, a resting state was used with the volunteer relaxed and with eyes open. During reading, the gamma coherences between the parietal and temporal cortex increased. In addition, alpha coherence also demonstrated an increase in temporal electrodes.

The study by Bedo et al.,^(^^24^^)^ analyzing independent components, showed that, both in the ventral occipitotemporal cortex and in the central cortex, progression of theta band and gamma band synchronization occurs, and the information flow from these bands are associated with the visual and language processing areas.

The study,^(^^25^^)^ carried out with two male volunteers, evaluated guitar score reading, involving an unpublished sequence of musical notes, previously developed for the experiment. The results obtained revealed changes in the theta to gamma bands during cognitive activities. Our study shows changes in the four brain rhythms during reading activities.

In a review by Klimesch,^(^^26^^)^ alpha activity is suppressed in the word reading situation, which was also evidenced in our present study.

In the research by Fitzgibbon et al.,^(^^27^^)^ an analysis implemented in 20 adults showed that, during cognitive tasks, including reading, the powers of the gamma and theta bands increased, respectively, by two to five, and one to three times, when compared to resting.

The study by Kornrumpf et al.,^(^^28^^)^ aimed to investigate the spatial distribution of attention during reading, analyzing alpha band oscillations in the EEG. In their findings, it was concluded that, during reading, there is a right lateralization of posterior alpha activity, which is directly linked to attention, and is also related to oculomotor behavior in reading.

The study by Bizas et al.,^(^^4^^)^ evaluated the EEG of 17 neurologically healthy, adult and right-handed volunteers, during the execution of several cognitive processes, involving reading. EEG records were quantified using energy spectrum measurements in the four frequency bands (delta to beta). The results led to the conclusion that the left cerebral hemisphere showed greater spectral variations in the word reading situation. In contrast, our study showed greater PCP variation in the right hemisphere for the parietal and occipital regions. In the other regions, the variations in both hemispheres were similar.

## CONCLUSION

This article is essentially focused on the reading activity and, therefore, the results characterize this pure cognitive process, avoiding several types of simultaneous stimuli and allowing the quantitative results to be associated specifically with the physiology of reading, based on the electroencephalographic signal.

Most of the results obtained in this research are in agreement with the literature. Particularly considering the faster, gamma (30-80Hz) and high-gamma (80-100Hz) rhythms, the changes in the power contribution percentage values in the temporal and occipital regions during the word reading situation, compared to the frontal and central regions, increased reasonably. The quantitative data obtained represent a proposal for a “normal” quantitative pattern associated with the pure reading process, in view of the high number of individuals evaluated. Therefore, these results can be useful both in the assessment of clinical neurological conditions (patients in a coma) and for brain–machine interface applications (patients/individuals with hearing/verbalization problems). For a patient in an open-eyed coma-like state, unable to communicate verbally, if brain changes similar to those found in this study are observed, this could be an indication that the patient can read. This may be an alternative communication channel between the patient and the medical team/family. However, it is impossible to affirm that the same changes found in a “normal” neurological situation are present in a neurological condition with any form of brain dysfunction. These perspectives represent possible future studies, as a result of this article.
