# Supplementary material for: Analysis of electroencephalography brain rhythms in the reading process
Source: Einstein (Sao Paulo). 2020 Oct 29;18:eAO5442. doi: 10.31744/einstein_journal/2020AO5442 (PMC7607920; doi:10.31744/einstein_journal/2020AO5442)
Supplement: Translation

# Translation: Análise dos ritmos cerebrais de eletroencefalografia no processo de leitura

**Authors:** Camila Davi Ramos, Izabella Nonato Oliveira Lima, Amanda Luiza Rodrigues, Kaliny Alice Carvalho de Oliveira Magalhães, Aurélia Aparecida de Araújo Rodrigues, João-Batista Destro-Filho

**Affiliations:** 1 UberlândiaMG Brasil Universidade Federal de Uberlândia, Uberlândia, MG, Brasil.

**Keywords:** Eletroencefalografia, Leitura, Ritmo gama, Ritmo delta, Ritmo beta

## Abstract

**Objetivo:**

Verificar se, por meio de três quantificadores distintos, o sinal eletroencefalográfico medido em repouso é diferente do sinal medido durante o processo de leitura, especialmente considerando os ritmos rápidos, gama e supergama, assim como ocorre nos ritmos clínicos delta a beta.

**Métodos:**

Foram avaliados 96 sinais eletroencefalográficos medidos em voluntários neurologicamente saudáveis, em dois momentos: repouso e leitura de palavras. Cada trecho do sinal foi mensurado por três quantificadores que medem, de maneira isolada, a potência normalizada e a potência percentual, bem como a coerência entre os hemisférios direito e esquerdo. O teste estatístico de Mann-Whitney foi usado para comparar os resultados dos quantificadores em cada faixa cerebral.

**Resultados:**

Os ritmos gama e supergama apresentaram comportamento mais distinto entre os momentos analisados (repouso e leitura) que os ritmos clinicamente analisados.

**Conclusão:**

Esse achado contribui com a escassa literatura segundo a qual os ritmos rápidos podem conter informações que normalmente são descartadas na neurologia clínica.

## INTRODUÇÃO

A leitura é responsável por aprimorar o armazenamento de informações no homem, sendo necessária uma conjuntura de habilidades e estudo contínuo para que a aprendizagem de novas palavras proporcione ampliação nos conhecimentos.^(^^1^^)^ A fim de que uma palavra seja reconhecida, múltiplos sistemas cerebrais precisam se conciliar, para que ocorra a interpretação homogênea da entrada visual.^(^^2^^)^ Pesquisas indicam que várias regiões cerebrais são acionadas durante a leitura,^(^^3^^)^ porém, por meio da neuroimagem, constata-se que uma área do córtex occipito-temporal é ativada em leitores fluentes.^(^^4^^)^ Essa região, localizada no giro fusiforme, foi nomeada de “área visual da palavra” (*visual word form area*).^(^^5^^)^ Estudos desenvolvidos demonstram que tal região da leitura ativa-se quando indivíduos são submetidos às palavras escritas, mas não se ativa quando as mesmas são ouvidas.^(^^5^^)^ A prática da leitura, além de levar ao aprimoramento dos conhecimentos prévios, também é responsável pelo aperfeiçoamento da percepção auditiva e visual,^(^^6^^)^ sendo uma forma particular da evolução humana.

Durante o processo de leitura, a atividade elétrica do cérebro sofre alterações, as quais podem ser captadas pelo exame eletroencefalográfico (EEG). Sabendo-se que a transmissão das informações cerebrais ocorre por meio de atividade elétrica, o EEG grava essa atividade, que é detectada por eletrodos colocados no couro cabeludo,^(^^7^^,^^8^^)^ conforme as seguintes localizações neuroanatômicas: frontal (F), central (C), parietal (P), temporal (T), occipital (O). Os eletrodos ímpares representam o hemisfério esquerdo cerebral, e os pares, o hemisfério direito.^(^^9^^)^

A análise espectral de EEG é realizada em suas bandas de frequência, as quais possuem limites bem definidos,^(^^9^^,^^10^^)^ sendo elas: delta (variando entre 0,5 e 3,5Hz), teta (entre 3,5 e 7,5Hz), alfa (que varia entre 7,5 e 12,5Hz), beta (entre 12,5 e 30Hz), gama (entre 30 e 80Hz) e supergama (acima 80Hz).^(^^10^^,^^11^^)^ A literatura afirma que os ritmos gama e supergama estão relacionados às tarefas cognitivas que envolvem percepção, atenção e memória.^(^^12^^,^^13^^)^ Todavia, do conhecimento dos autores, nenhum artigo encontrado analisou apenas o processo de leitura em si; todas as análises feitas em estudos anteriores a esse utilizavam juntamente a leitura com compreensão de palavras, a interpretação de texto e o estímulo visual. Além disso, poucos artigos estudam o processo de leitura em faixas de frequência acima de 40Hz,^(^^1^^,^^4^^,^^9^^,^^14^^)^ principalmente no contexto de indivíduos brasileiros ou latino-americanos, e avaliam o grau de modificação quantitativa do EEG entre repouso e processo de leitura, que é de suma importância para uma calibração correta da interface cérebro-máquina.

## OBJETIVO

Mensurar, por meio do processamento quantitativo, sinais eletroencefalográficos de voluntários neurologicamente saudáveis no processo de leitura, bem como em situação de repouso, e verificar se os ritmos gama e supergama contribuem majoritariamente na separação entre os momentos de leitura e de repouso; demonstrar quais são essas possíveis alterações na atividade cerebral que esses ritmos causam no cérebro nessas duas situações.

## MÉTODOS

Este estudo foi realizado no primeiro semestre do ano de 2019 e abordou os seguintes tópicos de processo metodológico: coleta de dados; pré-processamento; quantificação do sinal cerebral; e análise estatística.

### Coleta de dados

Sinais EEG considerados neurologicamente normais foram analisados, tendo sido retirados de uma base de dados de EEG e medidos em voluntários saudáveis, entre os anos de 2016 e 2018.^(^^15^^)^ A autorização pelo Comitê de Ética em Pesquisas (CEP) da Universidade Federal de Uberlândia (UFU) foi liberada sob número CAAE: 54781615.6.0000.5152, com parecer de número 1.715.960, permitindo a coleta e uso/processamento desses dados. Dessa base, foram utilizados 96 registros de EEG (49 referentes a voluntários do sexo masculino). A média de idade dos voluntários foi de 24±7,5 anos.

O equipamento operado para a coleta dos exames possuía capacidade para 20 canais, e a filtragem utilizada em todos os registros fez uso de filtro passa-alta com frequência de corte de 1Hz, filtro passa-baixa a 100Hz e filtro rejeita faixa entre 58 e 62Hz. A configuração utilizada no aparelho como frequência de amostragem e impedância foi de 1kHz e 1 KΩ, respectivamente. Todos os registros foram coletados seguindo o sistema internacional de coletas 10-20. O protocolo envolveu o registro de dois eventos: evento 1, denominado sem estímulo (SE), e evento 2, em que o voluntário foi submetido à leitura sequencial de dez palavras, aqui denominado leitura de palavras (LP).

Para a gravação SE, o voluntário permaneceu deitado, de olhos abertos e em repouso, ao longo de 3 minutos, sem nenhuma estimulação. Já em LP, com o voluntário ainda deitado, foram apresentadas uma palavra de cada vez, sequencialmente. Essas eram mostradas, durante um período de 5 segundos, na altura dos olhos do voluntário, escritas em tipos de letras grandes em um pedaço de cartolina, de forma que ele conseguisse fazer a leitura silenciosa. Posteriormente à leitura, realizaram-se mais 5 segundos de gravação, em silêncio, antes de passar para a próxima palavra. No total, o voluntário foi solicitado a ler dez palavras, a saber: cachorro, futebol, camisa, novela, banana, tomate, música, celular, tecido e trabalho. Essa escolha, embora tenha sido aleatória, buscou utilizar vocábulos de forma a evocar memória e emoções associadas ao dia a dia atual de um brasileiro comum. Um exemplo de um sinal filtrado e não filtrado de EEG no domínio do tempo, coletado em um voluntário para ambas as situações aqui estudadas, é apresentado na figura 1.

**Figura 1:** Exemplo de sinais não filtrados e filtrados de eletroencefalografia no domínio do tempo, coletados em um voluntário. (A) sinal não filtrado referente à situação sem estímulo; (B) sinal filtrado referente à situação sem estímulo; (C) sinal não filtrado referente à situação leitura de palavras; (D) sinal filtrado referente à situação leitura de palavras

### Pré-processamento

Para garantir que o estudo considerasse um intervalo de tempo durante o qual o voluntário realmente estivesse realizando o ato da leitura da palavra, foram excluídos o primeiro e o último segundo de cada registro das palavras lidas. Assim, para o evento LP, foram analisadas dez épocas (trechos e segmentos) com duração de 3 segundos, cada época associada à leitura de uma palavra específica. Já para o evento SE, cada registro de EEG foi submetido à validação de separação de épocas de interesse. As épocas, também com duração de 3 segundos de sinal, foram escolhidas por um médico neurologista, especialista em análise de EEG, por meio da visualização. Adotou-se como padrão para escolha a morfologia do traçado neurologicamente normal, evidenciando momentos livres de artefatos visuais, cujas morfologias das ondas cerebrais estivessem dentro dos padrões esperados para um voluntário saudável.

A fim de que o sinal analisado fosse o mais livre possível de artefatos, como ruído de 60Hz, de artefato muscular, de piscada, ou outros, foram utilizadas, além de análises visuais do traçado, filtragens específicas, o que garantiu que o sinal analisado fosse referente ao traçado EEG.

### Quantificação do sinal cerebral

A primeira ferramenta utilizada na quantificação do sinal foi a transformada de Fourier de tempo discreto (TDF), cujo cálculo resulta na densidade espectral (potência) do sinal distribuída em cada frequência analisada. O cálculo da TDF é definido como mostrado na equação 1.

(Equação 1) $$F(\omega )=\int_{-\infty }^{\infty }f(t)e^{-i\omega t}dt$$

onde F(ω) é a TDF de f(t).

O quantificador percentual de potência normalizada (PPN) mensura a quantidade de potência do sinal, abrangendo todos os ritmos existentes entre 1 e 100Hz (delta, teta, alfa, beta, gama e supergama). Já o quantificador percentagem de contribuição de potência (PCP) mensura, de maneira relativa, a potência presente em cada ritmo cerebral, em relação à potência total do espectro, informando a contribuição que cada ritmo tem perante a época avaliada. Os dados de entrada de ambos quantificadores foram dez épocas referentes ao evento SE e dez épocas referentes ao evento LP.

Tanto para PPN quanto para PCP, inicialmente calcula-se a densidade espectral de potência, *S*_x_, por meio da TDF.^(^^15^^–^^17^^)^ Com o resultado do cálculo de *S*_x_, feito para todas as épocas dos eventos SE e LP, foram calculados os valores de PPN (equação 2), bem como os valores de PCP (equação 3).^(^^18^^)^ O quantificador PPN é definido como na equação 2.

(Equação 2) $$PPN_{x_{a}}(f,b)=\frac{S_{x}a(f,b)}{Máx\left(S_{x}a\right)}$$

Em que S_x_a(f,b) é densidade espectral de potência do sinal EEG no eletrodo X, para o evento *a* (SE ou LP); em que *b* e *f* correspondem, respectivamente, à época e ao valor de frequência correspondentes. S_x_a é a matriz de densidade espectral de potência, em que as linhas representam os valores de frequências (f), e as colunas são referentes às épocas (b) [W/Hz]. Máx(S_x_a) é a amplitude máxima, calculada após análise de todos elementos da matriz S_x_a [W/Hz].

Desde modo, o cálculo do quantificador PCP é dado conforme a equação 3:

(Equação 3) $$PCP_{ritmo}i=\frac{\int_{f=f_{in}^{\;\;\;ritmo\;}}^{f_{\text{out }}ritmo\;}{\left|S_{x}i_{a}(f)\right|}^{2}df}{P_{i_{a}}}$$

Em que o índice *i* está associado às épocas; índice *a*, ao evento (SE ou LP); *f*_in_*ritmo* é o primeiro valor de frequência do ritmo analisado [Hz]; *f*_out_*ritmo* é o último valor de frequência do ritmo analisado [Hz]; *Pi*_a_ é a potência calculada em todo espectro [W], sendo *a* o evento.

Os dados de entrada para o quantificador coerência foram os mesmos que para PPN e PCP (épocas selecionadas de cada evento). A coerência é uma medida estatística relacionada ao grau de acoplamento dos espectros de frequência, e seu valor depende das correlações entre os eventos no domínio da frequência. Pode-se ainda fornecer informações adicionais em diagnósticos, ao se medirem os pares de regiões neocorticais.^(^^18^^–^^20^^)^ Refere-se ao grau de similaridade de fase entre dois sinais.^(^^21^^)^ O cálculo da coerência é feito pela magnitude quadrada da densidade espectral cruzada entre os dois sinais, como se observa na equação 4.

(Equação 4) $${\left|{\text{Γ}}_{xy}\left(e^{jw}\right)\right|}_{\;\;\;b}^{2}=\frac{{\left|S_{xy}\left(e^{jw}\right)\right|}^{2}}{S_{x}\left(e^{jw}\right)S_{y}\left(e^{jw}\right)}$$

Sendo I *b* a época considerada; S_xy_, a densidade espectral de potência cruzada [W/Hz]; S_x_, a densidade espectral do eletrodo X; e S_y_, a densidade espectral do eletrodo U.

Os valores resultantes da utilização equação 3 variam de zero a um, e valores próximos de zero apresentam baixa correlação, e aqueles próximos de um indicam correlação alta. Essa propriedade é expressa pela equação 5.

(Equação 5) $$0\leq {\left|{\text{Γ}}_{xy}\left(e^{jw}\right)\right|}^{2}\leq 1$$

Para a realização dos cálculos de coerência, foram levados em consideração os pares de eletrodos F3–F4, C3–C4, P3–P4 e O1–O2, considerando o espectro de 1 a 100Hz, abordando do ritmo delta ao ritmo supergama.

### Análise estatística

Como os valores de PPN variaram em uma faixa grande de frequências (1 a 100Hz), optou-se por repartir esses dados em três bandas: ritmos clínicos (variando entre 1 e 30Hz); ritmo gama (30 a 80Hz); e ritmo supergama (80 a 100Hz). A partir dos valores de PPN por banda, os eventos SE e LP foram dispostos nessa ordem, para que diagramas tempo-frequências pudessem ser elaborados. Tais valores foram dispostos no tempo em ordem cronológica de acontecimentos e na frequência, do menor ao maior valor (considerando cada banda isoladamente). Para cada eletrodo analisado, foram gerados três diagramas tempo-frequência: ritmos clínicos, ritmo gama e ritmo supergama.

A fim de verificar a hipótese da igualdade dos dados de PPN em ambos os eventos (SE e LP), o teste de Mann-Whitney, foi utilizado, com intervalo de confiança de 95%. Trata-se de teste não paramétrico responsável pela comparação de amostras independentes com o objetivo de testar se existe diferença entre elas.^(^^22^^)^ O resultado mostra o nível de significância entre os dados, representados pelo valor de p. Este, quando menor que 5%, rejeita-se a hipótese da igualdade, assumindo-se que os dados comparados são distintos.

Os valores de PCP obtidos foram submetidos ao cálculo descritivo (mediana±desvio padrão de mediana) para resumir as informações obtidas por cada ritmo cerebral. Estes também foram comparados para os dois eventos por meio do teste Mann-Whitney com mesmo nível de significância, e os resultados dispostos em tabela.

Por fim, os valores de coerência obtidos tanto no evento SE, quanto no evento LP, foram utilizados para compor diagramas tempo-frequência. O eixo tempo foi formado, assim como para PPN, por valores medidos nas épocas cronológicas, respeitando a ordem dos acontecimentos, em segundos, e o eixo das frequências compreendeu valores que foram da menor à maior contribuição dentro de cada ritmo, em Hz. A comparação estatística entre os valores de coerência medidos no evento SE com os obtidos no evento LP foi implementada também usando o teste de Mann-Whitney, com 5% de significância.

## RESULTADOS

Na figura 2, são apresentadas topografias não normalizadas com valores médios de PCP de todos os voluntários para todos os ritmos: clínicos (delta à beta), gama e supergama. Desse modo, é possível observar como ocorrem a distribuição e a variação dos ritmos na situação em repouso e no processo de leitura por todo o escalpo. Por meio da topografia não normalizada, cada ritmo contém sua escala própria, sendo essa construída utilizando o valor médio máximo e mínimo de potência que aquele ritmo em questão apresenta.

**Figura 2:** Topografias dos valores médios de percentagem de contribuição de potência de todos os voluntários. (A) topografias dos ritmos clínicos (delta à beta), gama e supergama para a situação sem estímulo; (B) topografias dos ritmos clínicos (delta à beta), gama e supergama para a situação leitura de palavras

Na figura 3, estão representados os diagramas tempo-frequência dos valores de PPN. Em cada figura, os eletrodos Fz, Cz, Pz e Oz foram apresentados. A figura 3, de A a D, refere-se aos ritmos clínicos; a figura 3, de E a H, ao gama; e a figura 3, I a L, ao supergama. Em todas as comparações realizadas pela estatística de Mann-Whitney entre SE (1 a 30 segundos) e LP (30 a 60 segundos), os valores de p foram menores que 0,05, apontando diferença estatística entre PPN medido na situação de repouso e o PPN mensurado durante o processo de leitura.

**Figura 3:** Diagrama tempo-frequência de percentagem de potência normalizada para ritmos cerebrais, sendo os 30 primeiros segundos referentes ao evento sem estímulo, e os 30 últimos segundos referentes ao evento leitura de palavras. (A) diagrama referente ao eletrodo Fz, ritmos clínicos; (B) diagrama referente ao eletrodo Cz, ritmos clínicos; (C) diagrama referente ao eletrodo Pz, ritmos clínicos; (D) diagrama referente ao eletrodo Oz, ritmos clínicos; (E) diagrama referente ao eletrodo Fz, ritmo gama; (F) diagrama referente ao eletrodo Cz, ritmo gama; (G) diagrama referente ao eletrodo Pz, ritmo gama; (H) diagrama referente ao eletrodo Oz, ritmo gama; (I) diagrama referente ao eletrodo Fz, ritmo supergama; (J) diagrama referente ao eletrodo Cz, ritmo supergama; (K) diagrama referente ao eletrodo Pz, ritmo supergama; (L) diagrama referente ao eletrodo Oz, ritmo supergama Md: mediana; Dp: desvio padrão; E: sem estímulo; LP: leitura de palavras.

Ao fazer a análise da figura 3, de A a D, notou-se que, na faixa de frequência entre 10 e 13Hz, a qual a banda alfa foi mais evidente, a discrepância entre os eventos SE e LP foi perceptível. Quando se observa a figura 9 de E a H, que se referiu ao ritmo gama, notou-se que os maiores valores de PPN estiveram concentrados nas faixas de 30 a 52Hz e que não apenas visualmente, mas quantitativamente, esses valores diminuíram no processo de LP (valor de p <0,05). Já a observação da figura 3 de I à L, referente ao ritmo supergama, permitiu inferir que, nessa faixa de frequência, houve melhor separação entre SE e LP, além de demonstrar que, assim como no ritmo gama, houve diminuição dos valores de PPN no momento LP.

Os valores de PCP de cada evento, juntamente de seus respectivos valores de p, para cada ritmo cerebral, são expostos nas tabelas 1 (delta a beta) e tabela 2 (gama e supergama), para o conjunto de eletrodos C3, Cz, C4, P3, Pz, P4, T5, T6, O1, Oz e O2.

**Tabela 1:** Valores de percentagem de contribuição de potência – ritmos clínicos (α=5%)

Eletrodos e situação		Delta	Teta	Alfa	Beta
C3					
	SE	40,9	19,7	11,2	9,1
	LP	51,3	18,8	7,8	7,6
	Valor de p	0	0,6	0	0
CZ					
	SE	40,8	23,3	11,4	8,2
	LP	53,3	22,4	7,5	6,6
	Valor de p	0	0,3	0	0
C4					
	SE	41,1	18,3	12,9	9,2
	LP	52,9	18,2	7,6	7,4
	Valor de p	0	0,8	0	0
P3					
	SE	40,7	18,3	13,1	9,3
	LP	49,9	18,3	8,7	8,2
	Valor de p	0	0	0	0
PZ					
	SE	41,5	16,0	13,2	9,1
	LP	52,6	19,7	8,5	7,8
	Valor de p	0	0	0	0
P4					
	SE	40,2	14,7	14,7	9,2
	LP	50,8	18,5	8,9	8,3
	Valor de p	0	0	0	0
T5					
	SE	39,9	15,6	13,3	9,8
	LP	49,5	17,6	8,6	9,8
	Valor de p	0	0	0	0,5
T6					
	SE	39,9	13,2	14,4	9,6
	LP	51,3	17,0	8,4	9,1
	Valor de p	0	0	0	0
O1					
	SE	35,7	12,4	15,3	11,1
	LP	47,9	18,4	9,6	9,9
	Valor de p	0	0	0	0
OZ					
	SE	37,1	13,1	15,2	10,5
	LP	48,8	17,9	9,9	9,6
	Valor de p	0	0	0	0
O2					
	SE	35,3	10,9	18,2	11,0
	LP	47,8	18,1	10,4	10,1
	Valor de p	0	0	0	0

Valor de p≥0,05 indica igualdade estatística.

SE: sem estímulo; LP: leitura de palavras.

**Tabela 2:** Valores de percentagem de contribuição de potência – ritmos rápidos (α=5%)

Eletrodos e situação		Gama	Supergama
C3			
	SE	3,8	0,5
	LP	3,5	0,4
	Valor de p	0	0
CZ			
	SE	3,5	0,4
	LP	3,1	0,4
	Valor de p	0	0
C4			
	SE	3,7	0,5
	LP	3,0	0,4
	Valor de p	0	0,1
P3			
	SE	3,6	0,5
	LP	3,6	0,5
	Valor de p	0,7	0,7
PZ			
	SE	3,5	0,4
	LP	3,3	0,4
	Valor de p	0.1	0,1
P4			
	SE	3,5	0,5
	LP	3,7	0,5
	Valor de p	0,1	0,3
T5			
	SE	3,8	0,5
	LP	4,3	0,5
	Valor de p	0	0
T6			
	SE	3,6	0,5
	LP	4,1	0,5
	Valor de p	0	0
O1			
	SE	3,7	0,5
	LP	4,1	0,5
	Valor de p	0	0
OZ			
	SE	3,6	0,5
	LP	3,9	0,5
	Valor de p	0	0
O2			
	SE	3,7	0,5
	LP	4,1	0,5
	Valor de p	0	0

Valor de p≥0,05: indica igualdade estatística.

SE: sem estímulo; LP: leitura de palavras.

Ao analisar os valores correspondentes ao cálculo do PCP, percebeu-se que, ao se tratar dos ritmos clínicos, comparando SE com LP, o ritmo delta apresentou aumento da potência em todos os eletrodos durante a LP. Por outro lado, tanto no ritmo alfa, quanto no beta, os valores de PCP diminuíram no processo de leitura. A variação da banda de frequência teta apresentou comportamento diferente de PCP, conforme a região mensurada. Por exemplo, nas regiões P, O e T, o PCP aumentou durante o processo de leitura, enquanto nas demais regiões esse índice diminuiu. Já em gama e supergama, os valores de PCP diminuíram no processo de leitura, exceto na região occipital e temporal. Foi possível destacar, ainda, que os ritmos gama e supergama, em termos de PCP, eram sensíveis à região cerebral, ou seja, notou-se que, na região parietal, não havia diferenciação entre os valores de PCP medidos no momento de repouso e no de leitura. Isso indicou que, para tais ritmos, analisar os eletrodos nessa região não foi eficaz para segmentação entre repouso e atividade cognitiva.

A análise do terceiro quantificador do sinal EEG, coerência, indicou que tanto para os ritmos clínicos (Figura 4), quanto para os ritmos gama e supergama (Figuras 5 e 6, respectivamente), a coerência entre os pares simétricos dos eletrodos foi maior durante o processo de leitura. A discrepância entre tais valores medidos no momento de repouso e o momento de leitura foi nitidamente maior para os ritmos rápidos, o que apontou, mais uma vez, para a importância desses ritmos serem clinicamente considerados pelos neurologistas durante suas análises.

**Figura 4:** Diagrama tempo-frequência de coerência para ritmos clínicos, sendo os 30 primeiros segundos referentes ao evento sem estímulo, e os 30 últimos segundos referentes ao evento leitura de palavras. (A) diagrama referente ao eletrodo Fz; (B) diagrama referente ao eletrodo Cz; (C) diagrama referente ao eletrodo Pz; (D) diagrama referente ao eletrodo Oz Md: mediana; Dp: desvio padrão; SE: sem estímulo; LP: leitura de palavras.

**Figura 5:** Diagrama tempo-frequência de coerência para gama, sendo os 30 primeiros segundos referentes ao evento sem estímulo, e os 30 últimos segundos referentes ao evento leitura de palavras. (A) diagrama referente ao eletrodo Fz; (B) diagrama referente ao eletrodo Cz; (C) diagrama referente ao eletrodo Pz; (D) diagrama referente ao eletrodo Oz Md: mediana; Dp: desvio padrão; SE: sem estímulo; LP: leitura de palavras.

**Figura 6:** Diagrama tempo-frequência de coerência para supergama, sendo os 30 primeiros segundos referentes ao evento sem estímulo, e os 30 últimos segundos referentes ao evento leitura de palavras. (A) diagrama referente ao eletrodo Fz; (B) diagrama referente ao eletrodo Cz; (C) diagrama referente ao eletrodo Pz; (D) diagrama referente ao eletrodo Oz Md: mediana; Dp: desvio padrão; SE: sem estímulo; LP: leitura de palavras.

## DISCUSSÃO

A revisão de Antonenko et al.,^(^^23^^)^ descreve dois estudos de caso sobre a carga cognitiva da leitura de hipertextos em 20 estudantes, além da leitura de multimídia analisando 38 estudantes. Os resultados do primeiro caso mostraram diminuição significativa na potência de alfa durante os 20 segundos de leitura e, no segundo caso, essa redução também foi notada na região frontal do cérebro.

No estudo desenvolvido por von Stein et al.,^(^^20^^)^ com 19 voluntários, analisou-se a coerência em três modalidades distintas: palavra escrita, apresentação auditiva e apresentação visual do objeto. Cada estímulo teve duração de 2 segundos, com intervalo de 2 a 3 segundos entre todas as condições, e, para efeito de comparação, foi utilizado o repouso com o voluntário relaxado e de olhos abertos. Durante a leitura, as coerências gama entre os córtices parietal e temporal aumentaram. Além disso, a coerência alfa também demonstrou aumento nos eletrodos temporais.

O estudo de Bedo et al.,^(^^24^^)^ por meio da análise de componentes independentes, mostrou que, tanto no córtex occipito-temporal ventral, quanto no central, ocorre progressão da sincronização da banda teta e gama, sendo que o fluxo de informação dessas bandas está associado às regiões responsáveis pelas partes visual e de processamento de linguagem.

O estudo^(^^25^^)^ realizado com dois voluntários masculinos avaliou a leitura de partituras de violão, envolvendo uma sequência de notas musicais inéditas, desenvolvidas previamente para o experimento. Os resultados obtidos revelaram que as bandas teta a gama sofrem alterações durante atividades cognitivas. Ao fazer uma análise geral dos dados obtidos no presente estudo, os quatro ritmos cerebrais dispuseram de alterações durante as atividades de leitura.

Na revisão de Klimesch,^(^^26^^)^ ficou notório que a atividade alfa é suprimida no processo de leitura, o que também foi evidenciado no presente estudo.

Na pesquisa de Fitzgibbon et al.,^(^^27^^)^ uma análise implementada em 20 adultos mostrou que, durante tarefas cognitivas, inclusive na leitura, as potências das faixas gama e teta aumentaram, respectivamente, de duas a cinco, e de uma a três vezes, quando comparadas ao repouso.

O estudo de Kornrumpf et al.^(^^28^^)^ teve como objetivo investigar a distribuição espacial da atenção durante a leitura, analisando as oscilações da banda alfa no EEG. Desse modo, em seus achados, concluiu-se que, durante a leitura, existe uma lateralização alfa posterior direita, que está diretamente ligada à atenção, e também guarda relação com o comportamento oculomotor durante a leitura.

O estudo de Bizas et al.^(^^4^^)^ avaliou o EEG de 17 voluntários neurologicamente saudáveis, adultos e destros, durante a execução de diversos processos cognitivos, envolvendo a leitura. Os registros de EEG foram quantificados usando medidas do espectro de energia nas quatro bandas de frequência (delta a beta). Os resultados levaram a concluir que o hemisfério cerebral esquerdo apresentou maiores variações espectrais no processo de leitura. Em contrapartida, o presente estudo obteve maiores variações de PCP no hemisfério direito para as regiões parietal e occipital, e, nas demais regiões, ambos os hemisférios apresentaram variações similares.

## CONCLUSÃO

O presente artigo está essencialmente focado na atividade de leitura e, portanto, os resultados caracterizam este processo cognitivo puro, evitando-se diversos tipos de estímulos simultâneos e permitindo associar os resultados quantitativos especificamente com a fisiologia da leitura, baseado no sinal eletroencefalográfico.

A maioria dos resultados obtidos nessa pesquisa está em concordância com a literatura. Particularmente considerando os ritmos rápidos, gama (30Hz a 80Hz) e supergama (80Hz a 100Hz), o comportamento observado para valores de percentagem de contribuição de potência nas regiões temporal e occipital durante o processo de leitura, se comparada às regiões frontais e centrais, aumenta razoavelmente. Ainda como contribuição deste trabalho, deve-se salientar que os dados quantitativos obtidos representam proposta de padrão quantitativo “normal” associado ao processo de leitura puro, tendo em vista a elevada quantidade de indivíduos avaliada. Assim, tais resultados podem ser úteis tanto para a clínica neurológica (pacientes em estado de coma), quanto para aplicações em interface cérebro-máquina (pacientes/indivíduos com problemas de audição/verbalização). Para um paciente em estado de coma, que permanece de olhos abertos, porém não consegue se comunicar verbalmente, se forem observadas alterações cerebrais próximas às encontradas nesse estudo, este poderia ser um indicativo de que esse paciente consegue ler. Trata-se de uma alternativa de comunicação entre paciente e equipe médica/familiares, porém não é possível afirmar que as mesmas alterações encontradas em uma situação neurológica “normal” são apresentadas em uma situação neurológica com alguma disfunção cerebral. Em vista disso, tais perspectivas representam possíveis trabalhos futuros, em consequência deste artigo.
